# Research on Credit Default Prediction Model Based on TabNet-Stacking

**DOI:** 10.3390/e26100861

**Published:** 2024-10-13

**Authors:** Shijie Wang, Xueyong Zhang

**Affiliations:** 1 School of Finance, Central University of Finance and Economics, Beijing 102206, China; sjwang@mail.nankai.edu.cn; 2 RURAL CREDIT COOPERATIVE OF HEBEI, Shijiazhuang 050024, China

**Keywords:** TabNet, stacking, credit risk, risk control

## Abstract

With the development of financial technology, the traditional experience-based and single-network credit default prediction model can no longer meet the current needs. This manuscript proposes a credit default prediction model based on TabNeT-Stacking. First, use the PyTorch deep learning framework to construct an improved TabNet structure. The multi-population genetic algorithm is used to optimize the Attention Transformer automatic feature selection module. The particle swarm algorithm is used to optimize the hyperparameter selection and achieve automatic parameter search. Finally, Stacking ensemble learning is used, and the improved TabNet is used to extract features. XGBoost (eXtreme Gradient Boosting), LightGBM (Light Gradient Boosting Machine), CatBoost (Category Boosting), KNN (K-NearestNeighbor), and SVM (Support Vector Machine) are selected as the first-layer base learners, and XGBoost is used as the second-layer meta-learner. The experimental results show that compared with original models, the credit default prediction model proposed in this manuscript outperforms the comparison models in terms of accuracy, precision, recall, F1 score, and AUC (Area Under the Curve) of credit default prediction results.

## 1. Introduction

With the advent of the big data era, FinTech (Financial Technology) [1] is rapidly advancing. Many traditional financial companies are gradually changing from focusing on the front desk and neglecting the back office to neglecting the front desk and focusing on the back office. The trend of traditional offline loans going online is becoming more and more obvious. At the same time, the development of big data and credit reporting systems has greatly reduced the actual cost for financial institutions to obtain relevant data. Therefore, how to timely and efficiently use and analyze relevant data to detect high-risk behaviors such as fraud, gang fraud, default, and bad debts that may occur among applicants and ensure the safety of funds is an urgent problem to be solved in the healthy development of credit.

Due to its high accuracy and ease of use, logistic regression has gradually extended its application to personal credit research and is increasingly used in risk control by Western financial institutions [2]. For instance, Beninel used the Logistic Regression model to establish a transfer learning model for predicting credit risk across different customer categories [3]. Amir E. Khandani applied machine-learning techniques to construct nonlinear, nonparametric forecasting models of consumer credit risk and constructed out-of-sample forecasts that significantly improve the classification rates of credit card holder delinquencies and defaults [4].

Mohammed Azhan used polynomial Naive Bayes, support vector machines, logistic regression, random forests, and shallow neural networks to predict customer loan defaults, employing the F1 score as a metric for model performance [5]. Shihao Gu used the empirical context of return prediction as a proving ground; verified trees and neural networks were most valuable for forecasting larger and more liquid stock returns and portfolios [6]. Hassan Raza used advanced machine learning models to predict stock prices in the Pakistani stock market using 27 technical indicators and evaluated the performance of four models—ANN (Artificial Neural Network), SVM, LSTM (Long Short Term Memory), and Random Forest—and highlighted the importance of technical indicators in making accurate predictions [7]. Xianzheng Zhou constructed DNN (Deep Neural Networks) models for equity-premium forecasting, and the forecasting performance of DNN models was enhanced by adding additional 14 variables selected from finance literature [8].

TabNet [9] is a novel neural network structure designed specifically for tabular data and published by Google in AAAI (Association for the Advancement of Artificial Intelligence) in 2021. TabNet inherits the advantages of tree methods in interpretability and sparse feature selection, as well as the advantages of DNN representation learning and end-to-end training, challenging the monopoly of tree models in the field of tabular data processing.

Despite the continuous development of machine learning algorithms, the performance of a single model has always been bottlenecked, prompting scholars to explore new ways to combine models. Therefore, ensemble learning has emerged to break through the ceiling. In the boosting direction, most models are based on decision trees, and algorithms such as GBDT (Gradient Boosting Decision Tree), XGBoost, LightGBM, and CatBoost have emerged one after another. Among them, XGBoost was first proven to have made great progress in classification quality, operating efficiency, and ease of use [10,11,12,13]. In the stacking direction, due to the unique multi-layer architecture and combination mechanism of this method, the classification effect of the combined model can often be significantly improved [14].

In traditional ensemble algorithms, Boosting family algorithms are typically employed as the first layer of the ensemble model, with Logistic serving as the second layer, thus resulting in a relatively simplistic ensemble structure. To explore novel combinations for credit default prediction models, overcome the limitations of current single models, and enhance the ability of financial institutions to identify potential risks of borrowers prior to lending, this manuscript conducts the following research.

This manuscript proposes a Stacking ensemble learning model based on TabNet and introduces several enhancements: a multi-population genetic algorithm to improve TabNet’s feature selection module, and a PSO (Particle Swarm Optimization) algorithm to optimize TabNet’s parameter selection. The improved TabNet is used to extract features, and XGBoost, LightGBM, CatBoost, KNN, and SVM are selected as the first-layer base learners. The first layer consists of various independent classifiers, while the second-layer meta-learner is chosen to reduce the impact of classification errors from individual classifiers on the final result. For the second-layer meta-learner, a model with strong generalization capability is required. XGBoost is selected due to its high accuracy, strong generalization, and robustness to outliers in various practical tasks. The model’s performance is tested on the Alibaba Cloud-Tianchi loan default prediction dataset, evaluating its precision, F1 score, and AUC.

This study has made contributions to existing literature in three aspects. Firstly, to our knowledge, we are the first person to predict credit defaults based on the TabNet ensemble learning model in a financial academic paper. Unlike most studies that focus on traditional econometric models, we introduce a nonlinear machine learning model to predict the probability of credit default. Secondly, our model achieved significant performance improvement through ensemble learning, which also validates the existing financial literature on ensemble learning. Finally, our experimental results indicate that the model proposed in this paper can be applied to practical credit risk management, helping financial institutions identify high-risk loan applicants or loan projects and providing a scientific decision-making basis for financial institutions to take measures in advance.

Section 2 of this manuscript introduces existing credit default prediction models applied. Section 3 presents the specific design of the TabNet Stacking credit default prediction model proposed in this manuscript. Section 4 is about experimental design and analysis of experimental results. Section 5 is the conclusion.

## 2. Credit Default Prediction Model

Credit default prediction models can significantly enhance credit risk management. These models primarily employ modern technological methods to effectively predict credit risk and are continuously optimized during their operation. In the field of personal credit risk assessment, scholarly research typically focuses on two main aspects: indicator selection and model construction. This manuscript concentrates on the model construction aspect of personal credit risk assessment.

### 2.1. Boosting Models

#### 2.1.1. XGBoost Model

XGBoost belongs to the Boosting family of models within ensemble learning and has gained widespread attention due to its exceptional efficiency and high prediction accuracy. Its excellent scalability makes it particularly effective in addressing large-scale data challenges in industrial applications.

The XGBoost model is a variant of the GBDT model. It enhances the original GBDT approach by performing a second-order Taylor expansion of the objective function and incorporating regularization terms to reduce overfitting. XGBoost is an additive model that combines the outputs of k base learners to produce the final model. After multiple iterations, the model at iteration t can be expressed as Equation (1).

(1) $${\hat{y}}_{i}^{\left(t\right)}=\sum_{k=1}^{t}f_{k}(x_{i})={\hat{y}}_{i}^{\left(t-1\right)}+f_{t}(x_{i})$$

The loss function of XGBoost is the sum of the loss function and the regularization term used to prevent model overfitting and control model complexity. The loss function in XGBoost is as Equation (2).

(2) $$L(\Phi )=\sum_{i}^{n}l({\hat{y}}_{i},y_{i})+\sum_{k}^{K}\Omega (f_{k})$$

In the above formula, i represents the *i*th sample in the total sample; n represents the total number of samples used when training the *k*th tree; K represents all the trees trained; L represents the error between the true value and the predicted value of the *i*th sample; $\sum_{k}^{K}\Omega (f_{k})$ is the regularization term, which represents the complexity of the K trees. The smaller the value of the model, the stronger the generalization ability of the model.

To address the challenges of solving complex loss functions, the loss function is approximated using a second-order Taylor expansion, which results in the Equation (3).

(3) $$Obj^{\left(t\right)}\approx \sum_{i=1}^{n}\left[l(y_{i},{\hat{y}}_{i}^{\left(t-1\right)})+g_{i}f_{t}(x_{i})+\frac{1}{2}h_{i}f_{t}^{2}(x_{i})\right]+\Omega (f_{t})+u$$

$g_{i}=\partial_{{\hat{y}}_{i}^{\left(t-1\right)}}l(y_{i},{\hat{y}}_{i}^{\left(t-1\right)})$ is the first-order gradient of the loss function; $h_{i}=\partial_{{\hat{y}}_{i}^{\left(t-1\right)}}^{2}l(y_{i},{\hat{y}}_{i}^{\left(t-1\right)})$ is the second-order gradient (Hessian) of the loss function; $f_{t}(x_{i})$ is the prediction of the new tree being added, which can be expanded to Equation (4); $\Omega (f_{t})$ is the regularization term controlling the model’s complexity, which can be expanded to Equation (5).

(4) $$f_{t}(x)=w_{q(x)},w\in R^{T},q:R^{d}\to \{1,2,\ldots ,T\}$$

(5) $$\Omega (f_{t})=\gamma T+\frac{1}{2}\lambda \sum_{j=1}^{T}w_{j}^{2}$$

In the above formula, T represents the number of leaf nodes,γ and λ are weight parameters that control the number of leaves, and $w_{j}$ is the value of the *j*th leaf node.

Traversing all samples to calculate the loss function is equivalent to transforming a single sample into a set of leaf nodes and then calculating the loss function of each leaf node. At this time, each leaf node contains multiple samples; simplifying the loss function to the form of a leaf node yields Equation (6).

(6) $$\begin{array}{ll} Obj^{\left(t\right)} & ≃\sum_{i=1}^{n}\left[g_{i}f_{t}(x_{i})+\frac{1}{2}h_{i}f_{t}^{2}(x_{i})\right]+\Omega (f_{t})\\ & =\sum_{i=1}^{n}\left[g_{i}w_{q(x_{i})}+\frac{1}{2}h_{i}w_{q(x_{i})}^{2}\right]+\gamma T+\frac{1}{2}\lambda \sum_{j=1}^{T}w_{j}^{2}\\ & =\sum_{j=1}^{T}[(\sum_{i\in I_{j}}g_{i})w_{j}+\frac{1}{2}(\sum_{i\in I_{j}}h_{i}+\lambda )w_{j}^{2}]+\gamma T \end{array}$$

In the above formula, $G_{j}=\sum_{i\in I_{j}}g_{i},H_{j}=\sum_{i\in I_{j}}h_{i}$, $G_{j}$ is the accumulation of the first-order partial derivatives of the samples in the *j*th leaf node, and $H_{j}$ is the accumulation of the second-order partial derivatives of the samples in the *j*th leaf node. Therefore, the objective function of XGBoost can be further simplified to Equation (7).

(7) $$Obj^{\left(t\right)}=\sum_{j=1}^{T}[G_{j}w_{j}+\frac{1}{2}(H_{j}+\lambda )w_{j}^{2}]+\gamma T$$

Using the formula for finding the maximum value of a quadratic equation, it can be found when $w_{j}^{*}=-\frac{G_{j}}{H_{j}+\lambda }$, the objective function is minimized. The final loss function of the XGBoost model is shown in Equation (8).

(8) $$Obj=-\frac{1}{2}\sum_{j=1}^{T}\frac{G_{j}^{2}}{H_{j}+\lambda }+\gamma T$$

This manuscript uses Scikit learn API (Application Programming Interface) for parameter settings. After parameter optimization, the final parameter settings for the model are as follows: The parameter for establishing the number of subtrees is n_estimators = 200; The tree depth parameter is max_depth = 6; The minimum sample weight in the child nodes is min_child_weight = 1; The proportion of the training set is subsample = 0.8; and the Learning rate is learning_rate = 0.07.

#### 2.1.2. LightBGM Model

LightGBM was proposed mainly to address the issues GBDT encounters when handling massive datasets, enabling GBDT to be applied more efficiently and effectively in industrial practices. LightGBM introduces the Histogram algorithm, the GOSS (Gradient-based One-Side Sampling), and the EFB (Exclusive Feature Bundling) algorithm [15,16,17], building on the foundation of XGBoost. By incorporating these three algorithms, LightGBM significantly reduces the complexity required to generate a leaf, thereby greatly reducing computation time.

The histogram algorithm discretizes continuous floating-point features into k integers to form bins; that is, the feature values are binned, and the gradients and number of bins are accumulated. When traversing the data, the discretized values are used as indexes to accumulate statistics in the histogram. After traversing once, the histogram accumulates the required statistics, and then the histogram is used to find the optimal split point. The number of bins is much smaller than the number of different sample values, so there are fewer split points to traverse after binning, which greatly reduces the amount of calculation.

The single-sided sampling gradient algorithm is based on the perspective of reducing samples because the samples with smaller gradients have little effect on reducing residuals, so the focus is on samples with higher gradients. Therefore, in the single-sided sampling gradient algorithm, the samples with smaller gradients are first randomly excluded, and then the remaining samples with higher gradients are used to calculate the information gain. The specific method of the single-sided sampling gradient algorithm is: first arrange the eigenvalues to be split according to the absolute value, select the first a% samples with the largest gradient, and then take b% of the remaining small gradient samples. When calculating the information gain, multiply the last b% by (1−a)/b to amplify the weight of this part of the samples.

The EFB algorithm is designed to reduce the number of features, thereby speeding up LightGBM. When the dataset contains many sparse features, the EFB algorithm bundles mutually exclusive features together, reducing the feature dimensionality and effectively decreasing the number of features used to construct the histogram. This further reduces computational complexity. If two features are not entirely mutually exclusive, the degree of exclusivity can be measured using a conflict ratio. When the conflict ratio is low, two features that are not completely mutually exclusive can still be bundled together. This reduces the feature dimensionality while maintaining prediction accuracy.

This manuscript uses Scikit learn API for parameter settings. After parameter optimization, the final parameter settings for the model are as follows: The parameter for establishing the number of subtrees is n_estimators = 200; The tree depth parameter is max_depth = 9; The minimum sample weight in the child nodes is min_child_weight = 8.8; Number of leaves is num_leaves = 235. Learning rate learning_rate = 0.05; L1 regularization coefficient is reg_alpha = 0.45; and the L2 regularization coefficient is reg_lambda = 5.68.

#### 2.1.3. CatBoost Model

CatBoost is a combination of Categorical and Boosting. Compared with XGBoost and LightGBM, CatBoost’s innovations are as follows: it introduces an algorithm that can automatically convert categorical features into numerical features and also uses combined categorical features and ranking boosting. After this series of optimizations, CatBoost perfectly solves the problems of categorical features, gradient bias, and prediction offset, reduces the occurrence of overfitting, and thus improves the accuracy and generalization ability of the model.

The innovative algorithm introduced by CatBoost can automatically convert categorical variables into numerical variables; that is, by counting the frequency of categorical variables and adding hyperparameters, new numerical variables are generated. It also processes categorical variables by adding prior distribution terms to Greedy TS (Greedy Target-based Statistics). In addition, CatBoost also introduces methods such as Holdout TS (Holdout Target-based Statistics), Leave-one-out TS (Leave-one-out Target-based Statistics), and Ordered TS (Ordered Targe-based Statistics) to improve Greedy TS. These methods can reduce the impact of noise and low-frequency categorical data on sample distribution [18]. The basic principle is shown in Equation (9).

(9) $${\hat{x}}_{k}^{i}=\frac{\sum_{j=1}^{p-1}[x_{\sigma_{j,k}}=x_{\sigma_{p,k}}]Y_{\sigma_{j}}+a\cdot p}{\sum_{j=1}^{p-1}[x_{\sigma_{j,k}}=x_{\sigma_{p,k}}]+a}$$

In the above formula, p is the added prior term, a represents the weight coefficient, which is generally greater than 0.

The premise of feature combination is that several categorical features can be combined to generate a new feature. However, the number of combined features will continue to grow with the number of categorical features in the sample, so it is unrealistic to consider all combinations in the algorithm. CatBoost uses a greedy strategy to consider combinations when generating new split points for the tree. When the tree is split for the first time, no combination will be considered. For the second split, CatBoost will combine all combinations of the tree, categorical features, and all categorical features in the sample, and then automatically convert the new categorical features into numerical features.

To address the issue of prediction shift caused by gradient bias, CatBoost introduces a novel algorithm called Ordered Boosting. This method aims to obtain unbiased gradient estimates by training a model $M_{i}$ for each sample $x_{i}$ using data that does not include $x_{i}$. This model provides an estimate of the gradient for the sample, which is then used to train the base learners to produce the final model. However, since Ordered Boosting requires training n different models, it significantly increases memory usage and complexity, making it impractical for real-world applications. To overcome this, CatBoost optimizes the Ordered Boosting algorithm during the tree-building phase by using two boosting modes: Ordered and Plain. This optimization helps balance the need for accurate gradient estimation with practical constraints on memory and computational efficiency.

This manuscript uses Scikit learn API for parameter settings. After parameter optimization, the final parameter settings for the model are as follows: The parameter for the number of training sessions is iterations = 300. The tree depth parameter is max_depth = 6; The maximum size of one hot encoding is one_hot_max_size = 1; Learning rate learning_rate = 0.03; and the L2 regularization coefficient is l2_leaf_reg = 3.

### 2.2. TabNet Model

TabNet is the first self-supervised deep learning model for tabular data. When the amount of unlabeled data are greater than the amount of labeled data, TabNet pre-training can significantly improve the effect of supervised learning, which is suitable for the scenario of lack of labeled data in credit default prediction in this article. From the structure of TabNet, it can be found that it has strong scalability and a lot of room for subsequent optimization and improvement, so this article decided to use TabNet as the initial model for training.

**Figure 1:**
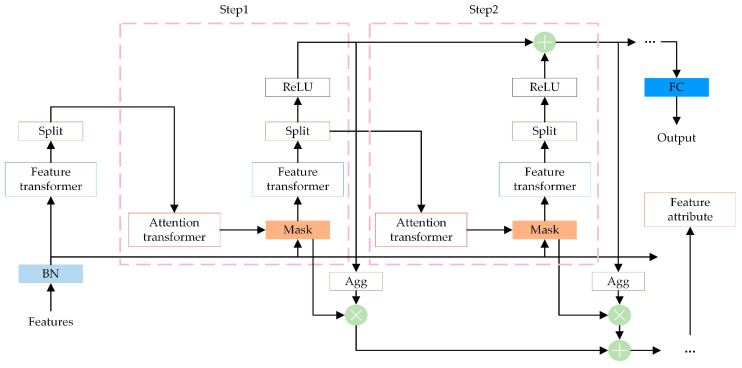
TabNet network structure.

The operation process of TabNet can be simply described as constructing the final feature vector through information aggregation after continuous feature selection to achieve the decision task. The TabNet network structure is shown in Figure 1.

**Figure 2:**
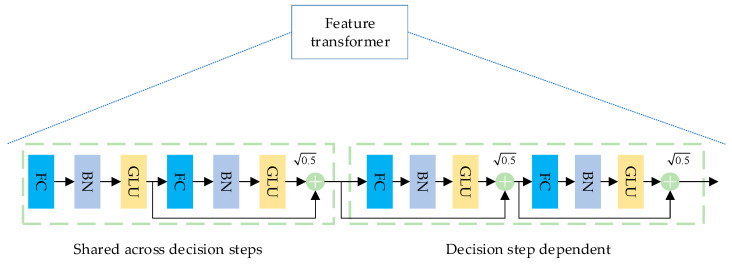
Feature Transformer layer structure.

TabNet as a whole can be considered as a multi-step additive model. The input data of each step model is the feature vector $F\in R^{B\times D}$, where B is the size of the batch size and D is the feature dimension. First, the original feature vector F is input to the BN (Batch Normalization) layer for normalization, and then the data passes through the Feature Transformer layer for feature calculation. The function of this layer is shown in Figure 2.

The Feature Transformer layer consists of a parameter-sharing layer and a parameter-independent layer. The parameter-sharing layer in the first half is trained together at all steps, and the parameter-independent module in the second half is trained separately at each step. This ensures that both the feature commonality and the feature characteristics can be calculated in the feature vector input at each step.

**Figure 3:**
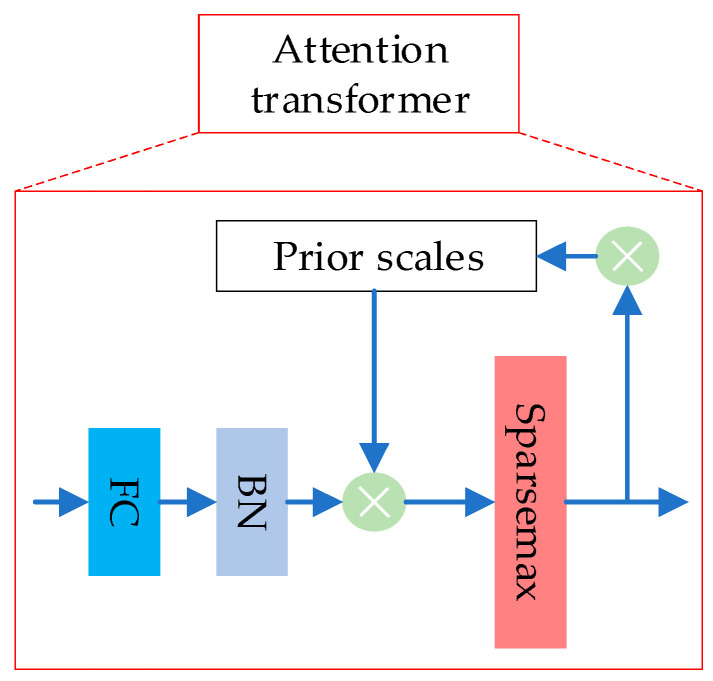
Attentive Transformer layer structure.

After passing through the Feature Transformer layer, the data will enter the Split module. The main function of this module is to divide the feature vector output by the Feature Transformer layer into two parts. One part participates in the calculation of the current step, and the other part is sent to the next layer to participate in the calculation of the Mask. When the data are first input into the model, Split does not split it and directly inputs the complete feature vector into the next layer. After passing through the Split module, the data enters the Attentive Transformer layer. The internal structure is shown in Figure 3.

The Attentive Transformer layer is mainly composed of the FC (Fully Connected) layer, BN layer, Sparsemax layer, and weighted scaling factor prior scale item. This layer will output a Mask matrix after calculation, and its mathematical expression is shown in Equation (10).

(10) $$M[i]=SparseMax(P[i-1]\cdot h_{i}(a[i-1]))$$

i represents the current step; a[i−1] is the feature vector after Split in the previous step, which is the feature information $B\times N_{a}$; $h_{i}(\cdot )$ represents the FC + BN layer; P[i−1] is the prior scale item of the previous step, which is used to indicate the degree of use of Features in previous decisions. If a feature has been used many times in the previous feature extraction process, it should not be selected by the model in the current feature selection. The main function of the prior scale item P[i] is to reduce the importance of such previously reused features in the current feature selection. Sparsemax is a sparse probabilistic activation function that works similarly to Softmax, but the output is more sparse, and the output results are concentrated near 0 or 1 with fewer intermediate values, which ensures that the most prominent features can be selected during the feature selection process. The formula for *P*[*i*] is shown in Equation (11).

(11) $$P[i]=\prod_{j=1}^{i}\left(\gamma -M[j]\right)$$

γ is the weight of the sparse regularization term, which is used to add constraints to the feature sparsity in the feature selection stage. The smaller γ is, the sparser the feature selection is. When γ=1 is used, it means that a feature item can only be used once in training; when γ>1 is used, it means that a feature can be reused in multiple steps of feature selection. As the number of uses increases, its weight value decreases, and the corresponding importance in subsequent feature selection decreases. When the model is initialized, the P[0] of all feature items is assigned a value of 1. In order to enhance the sparse selection capability of feature items, TabNet introduces a sparse regularization term in the form of entropy. The formula is shown in Equation (12).

(12) $$L_{sparse}=\sum_{i=1}^{N_{steps}}\sum_{b=1}^{B}\sum_{j=1}^{D}\frac{-M_{b,j}[i]}{N_{steps}\cdot B}\log(M_{b,j}[i]+\varepsilon )$$

N is the number of steps; B is the batchsize; D is the feature dimension, and is a small value, which is mainly used to stabilize the overall value. The main purpose of this regularization term is to make M[i] more it sparse, so that its distribution is closer to 0 or 1, and its value reflects the sparsity of M[j]. The smaller the value of $L_{sparse}$, the sparser M[j] is. Finally, $L_{sparse}$ will be added to the total loss function.

After the feature extraction of all steps is completed, TabNet transforms the output of the Feature Transformer layer of each step through the ReLU (Rectified Linear Unit) activation function, then adds the results of all steps together, and then passes through an FC layer to obtain the final output result, which is the category predicted by TabNet.

### 2.3. Stacking Ensemble Learning

Stacking is a more advanced model fusion method that leverages multiple base models for better predictions. Its core idea is to use several base learners in the first layer to learn from the original data. The outputs of these base learners are then stacked column-wise to form a new dataset. This new dataset is then passed to a second-layer model, known as the meta-learner, which fits the combined output to generate the final prediction. In simpler terms, stacking uses the output of the first layer as the input for the second layer, allowing the second-layer model to learn from the predictions of the base models and improve overall performance.

The primary issue with stacking is that the base models are trained on the entire training set, and then their predictions are used to fit the second-layer model, which often leads to overfitting. To address this problem, CV (Cross-Validation) is frequently used in practice to reduce overfitting. The following takes a five-fold cross-validation as an example to illustrate the working principle of Stacking in practical applications.

Step 1: Divide the training set into 5 parts, each of which is called a fold.

Step 2: For each fold, use it as a validation set and the remaining 4 folds as training sets, and use multiple base models to train the training set.

Step 3: For each base model i, use the trained model to predict the validation set, obtain the prediction result of the base model on the fold to obtain part of $P_{i}$, and use the model to predict part of $T_{i}$ in the test set.

Step 4: Repeat steps 2 and 3 until all 5 folds are used as validation sets to obtain corresponding predictions.

Step 5: Concatenate the predicted values of all fold validation sets into a complete $P_{i}$ as a new training set. At the same time, take the average of the prediction results of the five training models on the test set to obtain $T_{i}$.

Step 6: The $P_{i}$ generated by the i base models are merged to obtain the training set Train2 of the next layer, and the $T_{i}$ are merged to obtain the test set Test2 of the next layer.

Step 7: Use Train2 to train the second-layer model, and then obtain the prediction result on Test2, which is the final result of the model.

## 3. TabNet-Stacking Model

### 3.1. Improving TabNet Feature Selection

TabNet performs feature selection through a sequential attention mechanism similar to the additive model. This feature selection method inherits the advantages of the tree model’s interpretability and sparse feature selection, but TabNet still has room for improvement in the feature selection module.

Unlike other optimization algorithms, genetic algorithms operate on feature encoding rather than the features themselves, so there are many explanations for the features of the problem that do not need to be optimized. This feature can well solve the situation where the meaning of the features is unknown. At the same time, genetic algorithms have the advantages of efficient search, strong fault tolerance, and high flexibility. Therefore, this manuscript uses genetic algorithms to transform the attention module of TabNet. The pseudo-code of the genetic algorithm optimization feature selection algorithm is shown in Algorithm 1.
**Algorithm 1.** Genetic algorithm optimization feature selection algorithm pseudo-code.Input: Feature set $T=\{(x_{1},y_{1}),(x_{2},y_{2}),\ldots ,(x_{m},y_{m})\}$; Maximum genetic generation max; Population generation i;Initialization: i=0; max=500; Population $P_{i}$;while(i<max){  i++;  Perform selection operation on population $P_{i}$;  Perform crossover operation on population $P_{i}$;  Perform mutation operation on population $P_{i}$;}Output: Feature subset F


The encoding method used is binary, where 1 indicates that the feature vector is selected into the feature subset F, and 0 indicates it is not selected. The population is initialized randomly, and the fitness function is defined as the objective function of the TabNet model. The genetic operators are selected in sequence as roulette-wheel selection, single-point crossover, and basic bit mutation. To maximize the effectiveness of the genetic algorithm, several experiments were conducted, testing different numbers of generations, crossover probabilities, and mutation probabilities. The optimal settings were determined to be a maximum of 500 generations, a crossover probability $P_{c}$ of 0.7, and a mutation probability $P_{m}$ of 0.1, which provided the greatest improvement to the algorithm.

The genetic algorithm still has the problem of premature maturity when using a single population. This manuscript further uses multiple populations to replace the original single population. The operations between each population remain independent, and each generation of populations exchanges the excellent chromosomes of this generation with other populations to promote evolution. Among the multiple populations, one population focuses on the local optimal solution, and the other population focuses on the global optimal solution. Then, through immigration operations, the excellent chromosomes between populations are exchanged, so that the algorithm takes into account both local and global searches and strikes a balance between population diversity and algorithm convergence speed.

The specific improvement of the MPGA (Multi-Population Genetic Algorithm) over the standard genetic algorithm lies in the use of multiple populations, with each population configured with different parameters to evolve in various directions, thereby expanding the search space. MPGA introduces a migration operator, allowing information exchange between populations, which helps avoid population stagnation and accelerates convergence. Additionally, MPGA incorporates artificial selection and elite populations. The pseudo-code for the feature selection process using MPGA is presented in Algorithm 2.
**Algorithm 2.** Pseudo-code for Feature Selection using MPGA.Input: Feature set $T=\{(x_{1},y_{1}),(x_{2},y_{2}),\ldots ,(x_{m},y_{m})\}$; Maximum genetic generation max; Population generation i; Genetic generation r; the r-th population $P_{i}(r)$ in the i-th generation;Initialization: i=0; max=500;For r=1:MP   Initialize the population $P_{i}(r)$;  Calculate the $P_{i}(r)$ fitness of the population;while(i<max){  i++;For r=1:MP   Perform selection operation on population $P_{i}(r)$;  Perform crossover operation on population $P_{i}(r)$;  Perform mutation operation on population $P_{i}(r)$;  Calculate population fitness;Perform immigration operations on all populations $P_{i}$ in the i-th generation;Generating the elite population;}Select the best solution from the elite population to generate the best feature subset F;Output: Feature subset F


The multi-population optimization process operates as follows: the worst chromosome in the i−th population is replaced by the best chromosome from the (i−1)−th population using the migration operator. To maintain closure, the worst chromosome in the first population is also replaced by the best chromosome from the last population. The best chromosome from each population is then added to the elite population using the artificial selection operator.

### 3.2. Hyperparameter Optimization Based on Particle Swarm Optimization

**Figure 4:**
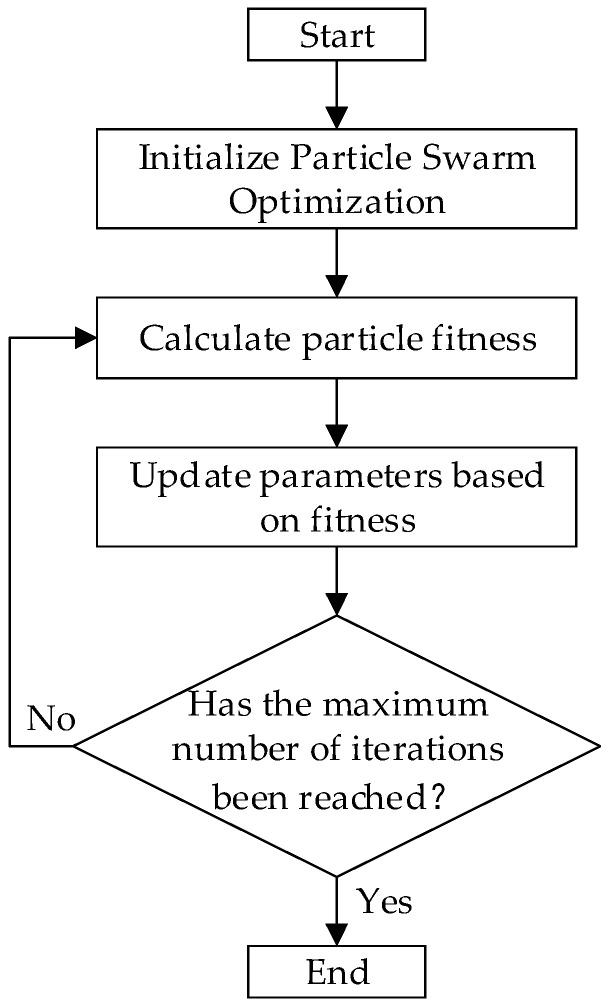
Particle Swarm Optimization for Hyperparameter Tuning.

Since most of TabNet’s operations are encapsulated in libraries, modifying its internal structure is challenging. Additionally, the complexity of TabNet’s parameters makes hyperparameter tuning difficult. Therefore, an automated method is needed to find the most suitable parameters for TabNet. Given that PSO is more efficient than Genetic Algorithms, we opted to use PSO for automatic hyperparameter tuning of TabNet. The process of hyperparameter optimization using PSO is shown in Figure 4.

Among them, the initial value of the weight factor w is set to 1, the initial values of the learning factor $c_{1}$ and the learning factor $c_{2}$ are both 0, the position of the initial particle is randomly generated, and the algorithm terminates after 500 iterations.

### 3.3. TabNet-Based Integrated Model

The TabNet model is a multi-step addition deep neural network model, and a large batch size is required to improve the model effect. TabNet consumes a lot of computing resources during the calculation process, which increases the system pressure in practical applications. Therefore, this manuscript uses the interpretability of the TabNet model as a feature selection module in the classification model, and the specific classification task is completed by the integrated learning model Stacking.

TabNet offers strong interpretability in feature selection, which sets it apart from other classification models. Traditional neural network classification models, such as those based on multilayer perceptrons, treat the input feature vectors uniformly across different layers of their internal network structure. In contrast, the TabNet model uses a Mask feature matrix to compute the features at each step, allowing it to assess the importance of individual features and thereby provide interpretability for the feature selection process.

Assuming that the feature vector is x, in the processing of step i, the output processed by the Feature Transformer layer is $d_{x}[i]\in R^{N_{d}}$. From the TabNet model structure, it can be seen that the final output of the model is obtained by adding the results of each step, and $d_{x,y}<0$ has no contribution to the entire model output. The contribution of the feature x[i] in step i can be obtained as Equation (13).

(13) $$\eta_{x}[i]=\sum_{j=1}^{N_{d}}ReLU(d_{x,j}[i])$$

The larger the $\eta_{x}[i]$ is, the greater the influence of the feature on the model output results and the greater the contribution to the entire model feature selection process. Therefore, $\eta_{x}[i]$ can be used as the weight of feature x[i] in the i−th step to weight the Mask matrix. The weight value in the Mask matrix reflects the importance of the feature, and the importance of each feature in the feature vector x can be defined as Equation (14).

(14) $$M_{agg-b,j}=\sum_{i=1}^{N}\eta_{x}[i]\cdot M_{x,j}[i]$$

The normalized importance is expressed as Equation (15).

(15) $$M_{agg-b,j}=\frac{\sum_{i=1}^{N}\eta_{x}[i]\cdot M_{x,j}[i]}{\sum_{j=1}^{D}\sum_{i=1}^{N}\eta_{x}[i]\cdot M_{x,j}[i]}$$

This process yields a feature importance ranking, achieving the goal of feature selection. In constructing the classification model, a two-layer Stacking ensemble method is employed to build the credit default classification model. First, after feature selection by TabNet, the student behavior dataset is split into five subsets using 5-fold cross-validation. Each subset is further divided into a sub-training set and a sub-validation set. The sub-training set is used to train base learners, generating lower-layer models, and the class predictions produced by the trained base learners on the validation set serve as inputs for the higher-level learner. The classification result of the higher-level learner is used as the final prediction of the model.

In the Stacking ensemble learning method, the greater the difference between the base learners, the better the overall performance of the model. Therefore, before building a Stacking model, it is necessary to analyze the differences between the various models. This manuscript selects the XGBoost model, LightGBM model, CatBoost model, KNN model, and SVM model as the base learners of the first layer of Stacking. Among them, the KNN model is mature in theory, has high accuracy, and is widely used in practical engineering problems. The MLP (Multilayer Perceptron) model has the characteristics of strong generalization ability and good stability and is an efficient classifier composed of artificial neural networks.

In the Stacking ensemble learning method, the greater the diversity among the base learners, the better the overall model performance. Therefore, before building the Stacking model, it is essential to analyze the differences between various models. In this study, XGBoost, LightGBM, CatBoost, KNN, and SVM models were selected as the base learners for the first layer of the Stacking model. Among them, the KNN model is theoretically well-established and highly accurate, making it widely applicable to real-world engineering problems. The MLP model, characterized by strong generalization ability and stability, serves as an efficient classifier composed of artificial neural networks.

**Figure 5:**
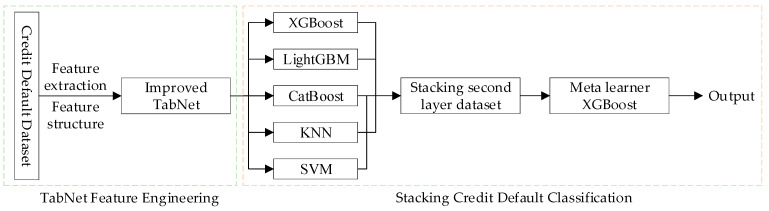
Credit default prediction model based on TabNet-Stacking.

The XGBoost, LightGBM, and CatBoost models belong to the Boosting family and are capable of flexibly handling various types of data, including both continuous and discrete data. They also allow the use of specific loss functions, enhancing robustness in dealing with outliers. The inclusion of KNN and SVM models ensures diversity among the algorithms in the Stacking model, which improves the overall classification performance. The first layer of the Stacking model consists of individual classification models, while the second layer’s meta-learner must minimize the classification errors of the base models to achieve a more accurate final result. For this purpose, a model with strong generalization capabilities is required. XGBoost, an ensemble learning model based on GBDT, has been optimized and offers high accuracy, strong generalization, and robustness against outliers in various tasks. Therefore, this study selects XGBoost as the meta-learner for the second layer. The structure of the credit default prediction model based on TabNet-Stacking is shown in Figure 5.

## 4. Experimental Results and Analysis

This experiment utilizes the open-source PyTorch framework, with the algorithm network implemented in Python (3.8.8). The hardware setup includes an NVIDIA RTX 4060Ti graphics card (8 GB) and a 64-bit Ubuntu 16.04 operating system.

PyTorch, an open-source neural network framework first released by Facebook in early 2017, directly supports models such as XGBoost, LightGBM, CatBoost, KNN, SVM, and TabNet. By leveraging open-source projects for these models and combining them with the preprocessed dataset from the Alibaba Cloud-Tianchi Loan Default Prediction dataset, the experiment optimizes feature selection using a multi-population genetic algorithm and fine-tunes hyperparameters using a particle swarm algorithm. The TabNet network structure is further improved, and the TabNet-Stacking model, consisting of Stacking ensemble learning, is applied to classify and predict customer data from the dataset, enhancing the credit risk management and prevention levels.

### 4.1. Data Set Processing

The data studied in this manuscript comes from the loan default prediction dataset in Alibaba Cloud Tianchi. The data comes from the loan records of a certain credit platform, totaling 800,000 records, containing 47 columns of variable information, of which 15 columns are anonymous variables. In this experiment, the dataset is divided into a training set and a test set in a ratio of 4:1 to train the model and evaluate the model performance.

In the data set, “id” is the unique letter of credit identifier assigned to the loan list, which is recorded as the index column in the model construction. “isDefault” is the customer label column, which takes a value of 0 or 1, where 1 indicates a defaulting customer and 0 indicates a non-defaulting customer. Among the remaining 45 variables, from the initial data storage type, there are 5 categorical variables, 8 discrete variables, 32 continuous variables, and 15 anonymous variables after desensitization are recorded as n0–n14. The specific meaning and explanation of each variable are shown in Table 1.

First, descriptive statistics are performed on numerical variables and categorical variables to understand the basic distribution of samples, as shown in Table 2 and Table 3.

In Table 2, count is the total number of variables, mean is the average value of the variable, std is the standard deviation, min is the minimum value, max is the maximum value, null is the null value, and entropy is the entropy value.

In the table above, conut represents the total number of variables, unique represents the type of variable, top represents the highest occurrence value, and freq represents the occurrence frequency of the top value. The last two columns in the table above are the number of missing values and the entropy value of the variable. The entropy value in statistics is also called information entropy. It is the most commonly used indicator of the purity of the observed sample set. The larger the value of information entropy, the greater the amount of information and the greater the contribution. Assuming that the proportion of the m−th class of samples in the sample set N is $p_{m}(m=1,2,3,\ldots ,M)$, the information entropy formula of the sample set N is shown in Equation (16).

(16) $$Ent(N)=-\sum_{m=1}^{M}p_{m}\log_{2}p_{m}$$

The entropy value of the variable “policyCode” is 0. By further checking the number of unique values of the variable, it is found that the unique value of “policyCode” is 1. It is determined that the variable has only one value and does not carry useful information for label classification, so it is deleted. The descriptive statistics show that 22 variables have missing values.

The highest missing rate does not exceed 10%. Since tree models are not highly sensitive to missing values, only simple analysis and imputation were performed. For the three variables “employmentTitle”, “postCode”, and “title”, which each have one missing value, the mode was used for imputation. For the variable “pubRecBankruptcies”, a comparison between the 0–1 distribution of missing data and normal data were conducted through visualization, revealing that the missing data’s distribution closely resembles that of 0. Therefore, missing values were filled with 0. For the missing variables “dti” and “revolUtil”, their counts were notably high when the label “isDefault = 1”, and based on entropy, these variables contain significant information and are important for the prediction results. Thus, missing values were imputed using the LGBMRegressor function, trained on non-missing data. For the variable “employmentLength”, the distribution of missing values differed from other categories, and with a missing rate of nearly 6%, the missing values were filled with -1 and treated as a separate category. Missing values in anonymized variables were relatively sparse and filled with 0.

### 4.2. Feature Selection

Feature selection is an extremely important task prior to formally constructing a model. In many machine learning algorithms, the original data typically contains a vast amount of information. However, much of this information may not be significant or useful for addressing specific problems. Thus, the objective of feature engineering is to select and extract features relevant to the problem and simultaneously remove redundant and useless features, thereby enhancing the performance and generalization ability of the algorithm.

For the variables “grade” and “subGrade”, both variables represent the loan grade status of customers. The value of “grade” gradually decreases from “A” to “G”. The value of “subGrade” is a subdivision under the value of “grade”, and each grade is further divided into 1–5 sub-grades. Therefore, “subGrade” is actually a further refinement of “grade”. The information contained in the two variables is repetitive, and “subGrade” has more information. Hence, only “subGrade” is retained for modeling. For the n-series variables, calculate the number of missing values, minimum value, maximum value, standard deviation, and average value of the n-series variables for each sample. Add five new variables, “n_null”, “n_min”, “n_max”, “n_std”, and “n_mean” for modeling.

Feature selection refers to selecting the most useful features from raw data for modeling, thereby improving the efficiency of modeling and also having a significant impact on the final effect of the model. By reducing some unnecessary features, there are benefits such as improving model accuracy, reducing overfitting risk, speeding up training, improving data visualization, and increasing model interpretability. The feature selection in this experiment uses the wrapper method, which, although computationally expensive, has higher accuracy compared to the filter method. Firstly, we fit the dataset based on the LightGBM classification model for the features that have undergone data cleaning and variable transformation and rank the importance of the features. The ranking results are shown in Table 4.

This manuscript uses LightGBM as the training model and employs the forward search method. Starting from the most important feature in Table 4, the model is added in sequence. When the number of features reaches 36, the model performs the best and receives the highest score. Although the feature importance of n7 and n10 is not significantly different, when the 37th variable n7 is added to the model, the performance of the model is not improved. Therefore, only the first 36 features are selected for modeling.

### 4.3. Evaluation Index

The credit risk control model is one of the most important applications in the financial industry. It is mainly used to assess the credit risk of borrowers and provide effective risk control and decision support for financial institutions. Since the performance of the model is directly related to the success or failure of the business, how to evaluate the performance of the model becomes a very important issue. Before constructing the indicators, the confusion matrix [19] is first introduced. Many evaluation indicators are constructed on this basis. The confusion matrix is shown in Table 5.

In order to quantitatively evaluate the comprehensive performance of the credit default prediction model based on TabNet-Stacking, this manuscript uses accuracy, precision, recall, F1 score, and AUC as evaluation criteria for credit default prediction.

Accuracy: Accuracy is the proportion of samples correctly predicted by the model to the total number of samples. In the credit risk control model, accuracy is defined as the proportion of the number of good and bad customers correctly predicted by the model to the total number of samples [20], as shown in Equation (17).

Precision: Precision is the proportion of samples predicted as positive to those that are actually positive. In the credit risk control model, precision is defined as the proportion of samples predicted as good to those that are actually excellent [21], as shown in Equation (18).

Recall: Recall is the proportion of samples predicted as positive to those that are actually positive. In the credit risk control model, recall is defined as the proportion of samples predicted as good to those that are predicted as excellent by the model [22], as shown in Equation (19).

F1-Score: The F1-Score is the harmonic mean of the precision and recall, taking into account the impact of both. In the credit risk control model, the F1-Score is defined as the harmonic mean of the model’s precision and recall, as shown in Equation (20).

(17) $$Accuracy=\frac{TP+TN}{TP+NP+TN+FN}$$

(18) $$Precision=\frac{TP}{TP+FP}$$

(19) $$Recall=\frac{TP}{TP+FN}$$

(20) $$F1=2\times \frac{Precision\times Recall}{Precision+Recall}$$

ROC Curve (Receiver Operating Characteristic Curve): The ROC curve is a two-dimensional plot with the FPR (False Positive Rate) on the x-axis and the TPR (True Positive Rate) on the y-axis. In credit risk control models, the ROC curve is used to evaluate the classification performance of the model and to assist in selecting the decision threshold [23]. The expressions of FPR and TPR are shown in Equations (21) and (22).

(21) $$TPR=Recall=\frac{TP}{TP+FN}$$

(22) $$FPR=\frac{FP}{FP+TN}$$

With the false positive rate FPR as the horizontal axis and the true positive rate TPR as the vertical axis, the ROC curve is introduced to measure the impact of different classification thresholds on the model classification results. The area under the ROC curve is called the AUC value. Generally, the closer the AUC value of the model is to 1, the better the classification effect of the model will be.

### 4.4. Analysis of Results

**Figure 6:**
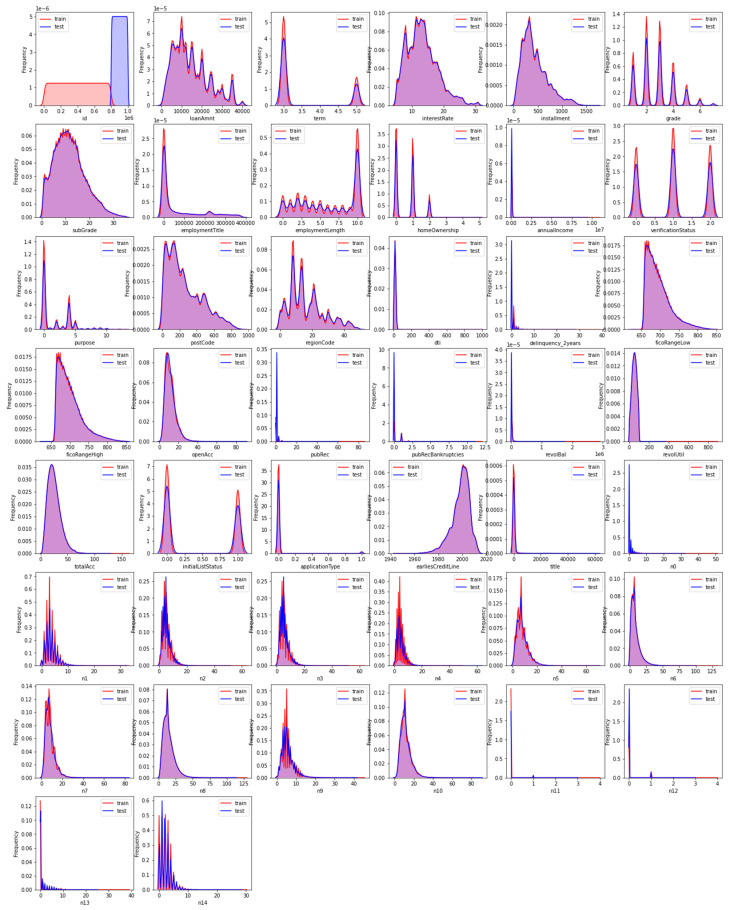
Training set and test set feature distribution.

This manuscript selects XGBoost, LightBGM, CatBoost, and TabNet models as comparison models. The data set is divided into training set and test set in a ratio of 4:1. After feature selection by the improved TabNet network, the feature distribution of the training set and the data set is shown in Figure 6.

The “id” column exhibited inconsistent feature distributions between the training and test sets, so this column was removed from the dataset. After training on the Alibaba Cloud-Tianchi Loan Default Prediction dataset, the performance of the TabNet-Stacking-based credit default prediction model and the comparison models were tested. The accuracy, precision, recall, F1 score, and AUC are shown in Table 6.

As can be seen from the table, compared with XGBoost, LightBGM, CatBoost, and TabNet models, the TabNet-Stacking model in this manuscript has the best performance in accuracy, precision, recall, F1 score, and AUC. The accuracy rate is improved by 2.19%~6.41%, the precision rate is improved by 8.91%~54.85%, the recall rate is improved by 7.94%~23.88%, the F1 score is improved by 8.36%~40.14%, and the AUC is improved by 6.57%~15.32%.

The accuracy rate is 0.021 higher than the best value of TabNet in the comparison model. In actual production, the accuracy of the model is generally accurate to 0.01. At this accuracy, the two models perform basically the same, indicating that the model as a whole can make a correct judgment on whether the user will default. The precision, recall, and F1 score have extremely remarkable improvements compared with the comparison models. Compared with XGBoost, they are increased by 0.277, 0.165, and 0.234, respectively. The recall rate is expressed as the proportion of customers that can be identified by the model in the case of real default. This is an important evaluation indicator in the field of anti-fraud. The highest F1 score also proves that the model in this manuscript has the best performance compared with the comparison model. The larger the AUC value, the better the performance of the model classification prediction and the more accurate the classification. The AUC value of the model in this manuscript reached 0.941, which is significantly higher than other models. In particular, the final model output of the solution uses the XBGoost model. Compared with the single XBGoost model without Stacking, the AUC value is 0.816, which is significantly improved, further illustrating the role of the TabNet-Stacking model in this manuscript in improving the performance of credit default classification.

The experimental results prove the practical value of the credit default prediction model based on TabNet-Stacking in this manuscript. Financial institutions can better evaluate the credit status of borrowers through this model, thereby reducing the risk of loan defaults. This helps to reduce the losses of financial institutions and protect the interests of investors. These findings are of great significance to bank credit analysts and risk control managers and provide guidance for using machine learning to improve credit granting decisions.

## 5. Conclusions

In order to improve the accuracy of feature extraction and classification of credit default prediction, this manuscript proposes a credit default prediction model based on TabNet-Stacking. Using the PyTorch (2.4.0) deep learning framework, the TabNet network model structure is improved, and a multi-population genetic algorithm is added to the network design to improve the feature selection module of TabNet, and the particle swarm algorithm is used to optimize the parameter selection of TabNet. Finally, in order to further improve the performance of model classification and prediction, Stacking ensemble learning is adopted, and the improved TabNet is applied to extract features. XGBoost, LightGBM, CatBoost, KNN, and SVM are selected as the first-layer base learners, and XGBoost is used as the second-layer meta-learner. The accuracy, F1 and AUC of the model are tested in the Alibaba Cloud-Tianchi loan default prediction dataset.

The experimental results demonstrate that, in comparison with XGBoost, LightGBM, CatBoost, and TabNet models, the credit default prediction model based on TabNet-Stacking proposed in this manuscript exhibits superior performance in predicting whether loan customers have default risks. This analysis based on TabNet-Stacking offers valuable insights into the interaction between machine learning models and key market features. However, the limitation of this study lies in the slight imbalance of the Alibaba Cloud-Tianchi loan default prediction data set. There is still room for further improvement in feature extraction. Moreover, out-of-sample prediction that might be helpful for enhancing prediction accuracy has not been explored. Additionally, the construction of integrated networks also merits more in-depth research. In the future, the directions of feature extraction and integrated network construction will be further integrated.

## Figures and Tables

**Table 1:** Dataset field dictionary.

Feature	Feature Meaning Explanation	Dtype
id	Unique letter of credit identifier assigned to the loan statement	int64
isDefault	Whether default	int64
loanAmnt	Loan Amount	float64
term	Loan term (year)	int64
interestRate	Loan interest rate	float64
installment	Installment amount	float64
grade	Loan grade	int64
subGrade	Loan subgrade	int64
employmentTitle	Employment title	float64
employmentLength	Employment length	float64
homeOwnership	Home ownership status	int64
annualIncome	Annual income	float64
verificationStatus	Verification Status	int64
issueDate	The month the loan was disbursed	object
purpose	Loan Purpose Category	int64
postCode	The first 3 digits of the ZIP code provided on your loan application	float64
regionCode	Region code	int64
dti	Debt-to-income ratio	float64
Delinquency_2years	The number of default events in the borrower’s credit file that are overdue for more than 30 days in the past two years	float64
ficoRangeLow	The lower limit range of fico that the borrower belongs to at the time of loan issuance	float64
ficoRangeHigh	The upper limit range of the borrower’s fico at the time of loan issuance	float64
openAcc	The number of open credit lines in the borrower’s credit file	float64
pubRec	Number of derogatory public records	float64
pubRecBankruptcies	Number of public record expungements	float64
revolBal	Total credit revolving balance	float64
revolUtil	Revolving credit utilization or the borrower’s use of all available revolving credit	float64
totalAcc	The total number of credit lines currently on the borrower’s credit file	float64
initialListStatus	Initial listing status of the loan	int64
applicationType	Individual application or joint application with two co-borrowers	int64
earliesCreditLine	The month in which the borrower’s earliest reported credit line was opened	int64
title	The name of the loan provided by the borrower	float64
policyCode	Publicly available strategy_code = 1 New product not publicly available strategy_code = 2	float64
n series of anonymous features	Anonymous features n0-n14, for processing some lender behavior counting features	float64

**Table 2:** Descriptive statistics for numerical variables.

Feature	Count	Mean	Std	Min	Max	Null	Entropy
loanAmnt	800,000	14,416.8	8716.09	500	40,000	0	6.947
term	800,000	3.483	0.856	3	5	0	0.797
interestRate	800,000	13.238	4.766	5.31	30.99	0	7.534
installment	800,000	437.948	261.460	15.69	1715.42	0	13.993
employmentTitle	799,999	72,005.4	106,585.6	0	378,351	1	14.037
homeOwnership	800,000	0.614	0.676	0	5	0	1.383
annualIncome	800,000	76,133.9	68,947.5	0	10,999,200	0	8.273
verificationStatus	800,000	1.010	0.783	0	2	0	1.576
purpose	800,000	1.746	2.367	0	13	0	1.936
postCode	799,999	258.536	200.037	0	940	1	8.897
regionCode	800,000	16.386	11.037	0	50	0	4.826
dti	799,761	18.285	11.150	−1	999	239	11.737
delinquency_2years	800,000	0.318	0.880	0	39	0	1.008
ficoRangeLow	800,000	696.204	31.866	630	845	0	4.357
ficoRangeHigh	800,000	700.204	31.867	634	850	0	4.357
openAcc	800,000	11.598	5.475	0	86	0	4.337
pubRec	800,000	0.215	0.606	0	86	0	0.807
pubRecBankruptcies	799,595	0.134	0.377	0	12	405	0.590
revolBal	800,000	16,228.71	22,458.0	0	2,904,836	0	15.173
revolUtil	799,469	51.791	24.516	0	892.3	531	9.879
totalAcc	800,000	24.999	11.999	2	162	0	5.520
initialListStatus	800,000	0.417	0.493	0	1	0	0.980
applicationType	800,000	0.019	0.137	0	1	0	0.137
title	799,999	1754.11	7941.47	0	61,680	1	3.969
policyCode	800,000	1.000	0.000	1	1	0	0.000
n0	759,730	0.512	1.333	0	51	40,270	1.292
n1	759,730	3.642	2.247	0	33	40,270	3.040
n2	759,730	5.643	3.303	0	63	40,270	3.564
n3	759,730	5.643	3.303	0	63	40,270	3.564
n4	766,761	4.736	2.950	0	49	33,239	3.382
n5	759,730	8.108	4.799	0	70	40,270	4.120
n6	759,730	8.576	7.401	0	132	40,270	4.519
n7	759,730	8.283	4.562	0	79	40,270	4.034
n8	759,729	14.622	8.125	1	128	40,271	4.877
n9	759,730	5.592	3.216	0	45	40,270	3.542
n10	766,761	11.644	5.484	0	82	33,239	4.339
n11	730,248	0.001	0.030	0	4	69,752	0.009
n12	759,730	0.003	0.062	0	4	40,270	0.032
n13	759,730	0.089	0.509	0	39	40,270	0.394
n14	759,730	2.179	1.844	0	30	40,270	2.706

**Table 3:** Descriptive statistics of categorical variables.

Feature	Count	Unique	Top	Freq	Null	Entropy
grade	800,000	7	B	233,690	0	2.328
subGrade	800,000	35	C1	50,763	0	4.635
employmentLength	753,201	11	10+ years	262,753	46,799	3.060
issueDate	800,000	139	2016/3/1	29,066	0	6.228
earliesCreditLine	800,000	720	Aug-01	5567	0	8.420

**Table 4:** Feature importance ranking.

Rank	Feature	Importance	Rank	Feature	Importance
1	issueDate	0.092667	26	title	0.016667
2	revolBal	0.064667	27	n_null	0.015667
3	annualIncome	0.057333	28	purpose	0.009667
4	loanAmnt	0.053333	29	delinquency_2years	0.008
5	regionCode	0.053333	30	n5	0.007
6	dti	0.044333	31	n_mean	0.005
7	homeOwnership	0.039	32	verificationStatus	0.005
8	subGrade	0.037	33	pubRec	0.005
9	installment	0.036667	34	n4	0.004667
10	earliesCreditLine	0.035333	35	applicationType	0.004667
11	employmentTitle	0.034667	36	n10	0.004667
12	term	0.034667	37	n7	0.004
13	interestRate	0.030333	38	n1	0.003667
14	m2	0.029	39	n0	0.002667
15	totalAcc	0.028	40	openAcc	0.002
16	n2	0.028	41	pubRecBankruptcies	0.001667
17	employmentLength	0.027667	42	n_std	0.001667
18	revolUtil	0.027667	43	n_max	0.001333
19	n14	0.026333	44	n_min	0.000333
20	n9	0.024	45	n13	0.000333
21	postCode	0.020333	46	m1	0.000333
22	ficoRangeHigh	0.02	47	initialListStatus	0.000333
23	ficoRangeLow	0.017667	48	n12	0
24	n8	0.017	49	n11	0
25	n6	0.016667			

**Table 5:** Confusion matrix.

Category	Predicting Default	Predicting No Default
Actual Default	TP	FN
Actual no default	FP	TN

**Table 6:** Overall data set algorithm comparison.

Model	Accuracy	Precision	Recall	F1-Score	AUC
XGBoost	0.920	0.505	0.691	0.583	0.816
LightBGM	0.924	0.523	0.701	0.599	0.822
CatBoost	0.951	0.677	0.738	0.702	0.854
TabNet	0.958	0.718	0.793	0.754	0.883
TabNet-Stacking	0.979	0.782	0.856	0.817	0.941

## Data Availability

The data that support the findings of this study are available from the Alibaba Cloud—Tianchi at https://tianchi.aliyun.com/competition/entrance/531830/information (accessed on 22 September 2024).

## Abbreviations

XGBoost	eXtreme Gradient Boosting
LightGBM	Light Gradient Boosting Machine
CatBoost	Category Boosting
KNN	K-NearestNeighbor
SVM	Support Vector Machine
AUC	Area Under the Curve
FinTech	Financial Technology
ANN	Artificial Neural Network
LSTM	Long Short Term Memory
DNN	Deep Neural Networks
AAAI	Association for the Advancement of Artificial Intelligence
GBDT	Gradient Boosting Decision Tree
PSO	Particle Swarm Optimization
API	Application Programming Interface
GOSS	Gradient-based One-Side Sampling
EFB	Exclusive Feature Bundling
Greedy TS	Greedy Target-based Statistics
Holdout TS	Holdout Target-based Statistics
Leave-one-out TS	Leave-one-out Target-based Statistics
Ordered TS	Ordered Targe-based Statistics
BN	Batch Normalization
FC	Fully Connected
ReLU	Rectified Linear Unit
CV	Cross-Validation
MPGA	Multi-Population Genetic Algorithm
MLP	Multilayer Perceptron
ROC Curve	Receiver Operating Characteristic Curve
FPR	False Positive Rate

## References

[1] Hu W., Li X. (2023). Financial technology development and green total factor productivity. Sustainability.

[2] Zhang W., Wang J. (2024). Credit risk contagion in complex companies network–Empirical research based on listed agricultural companies. Econ. Anal. Policy.

[3] Beninel F., Bouaguel W., Belmufti G. (2012). Transfer learning using logistic regression in credit scoring. arXiv.

[4] Khandani A.E., Kim A.J., Lo A.W. (2010). Consumer credit-risk models via machine-learning algorithms. J. Bank. Financ..

[5] Azhan M., Meraj S. (2021). Credit card fraud detection using machine learning and deep learning techniques. Proceedings of the 2020 3rd International Conference on Intelligent Sustainable Systems (ICISS).

[6] Gu S., Kelly B., Xiu D. (2020). Empirical asset pricing via machine learning. Rev. Financ. Stud..

[7] Raza H., Akhtar Z. (2024). Predicting stock prices in the Pakistan market using machine learning and technical indicators. Mod. Financ..

[8] Zhou X., Zhou H., Long H. (2023). Forecasting the equity premium: Do deep neural network models work?. Mod. Financ..

[9] Arik S.Ö., Pfister T. Tabnet: Attentive interpretable tabular learning. Proceedings of the AAAI Conference on Artificial Intelligence.

[10] Chen T., Guestrin C. Xgboost: A scalable tree boosting system. Proceedings of the 22nd Acm Sigkdd International Conference on Knowledge Discovery and Data Mining.

[11] Berhane T., Melese T., Seid A.M. (2024). Performance Evaluation of Hybrid Machine Learning Algorithms for Online Lending Credit Risk Prediction. Appl. Artif. Intell..

[12] Zedda S. (2024). Credit scoring: Does XGboost outperform logistic regression? A test on Italian SMEs. Res. Int. Bus. Finance.

[13] Hou L., Bi G., Guo Q. (2025). An improved sparrow search algorithm optimized LightGBM approach for credit risk prediction of SMEs in supply chain finance. J. Comput. Appl. Math..

[14] Yin W., Kirkulak-Uludag B., Zhu D., Zhu Z. (2023). Stacking ensemble method for personal credit risk assessment in Peer-to-Peer lending. Appl. Soft Comput..

[15] Álvarez Chaves M., Gupta H.V., Ehret U., Guthke A. (2024). On the Accurate Estimation of Information-Theoretic Quantities from Multi-Dimensional Sample Data. Entropy.

[16] Wang F., Zhang X., Liu L., Chen C., He X., Zhou Y. (2024). High-Precision Direction of Arrival Estimation Based on LightGBM. Circuits Syst. Signal Process..

[17] Liu X., Zhou B., Qi W., Wang J. (2024). Service Pricing and Charging Strategy for Video Platforms Considering Consumer Preferences.

[18] Hancock J.T., Khoshgoftaar T.M. (2020). CatBoost for big data: An interdisciplinary review. J. Big Data.

[19] Olaniran O.R., Alzahrani A.R.R., Alzahrani M.R. (2024). Eigenvalue Distributions in Random Confusion Matrices: Applications to Machine Learning Evaluation. Mathematics.

[20] Wang C., Chen B., Liu X. (2024). Credit diversification and banking systemic risk. J. Econ. Interact. Coord..

[21] Javadi S., Osah T. (2021). Credit risk correlation and the cost of bank loans. Financ. Manag..

[22] Song Y., Wang Y., Ye X., Zaretzki R., Liu C. (2023). Loan default prediction using a credit rating-specific and multi-objective ensemble learning scheme. Inf. Sci..

[23] Lu Z., Li H., Wu J. (2024). Exploring the impact of financial literacy on predicting credit default among farmers: An analysis using a hybrid machine learning model. Borsa Istanb. Rev..

